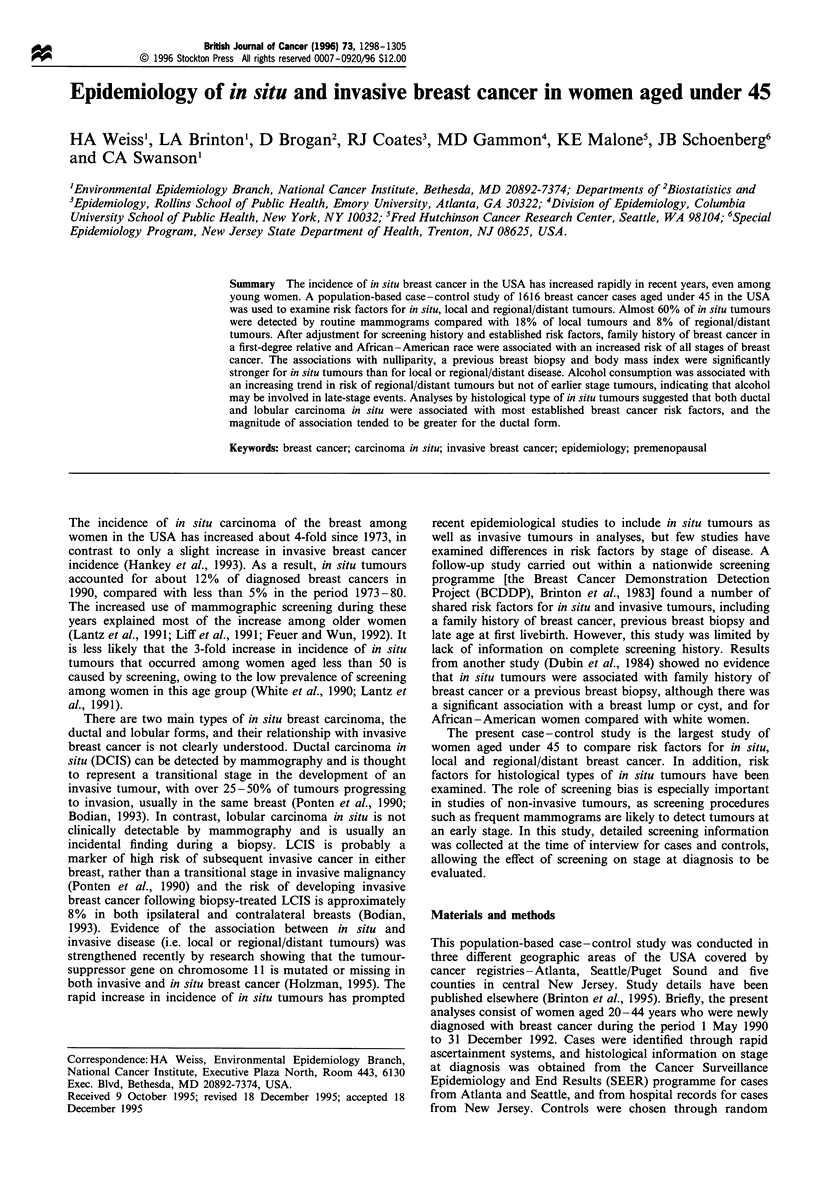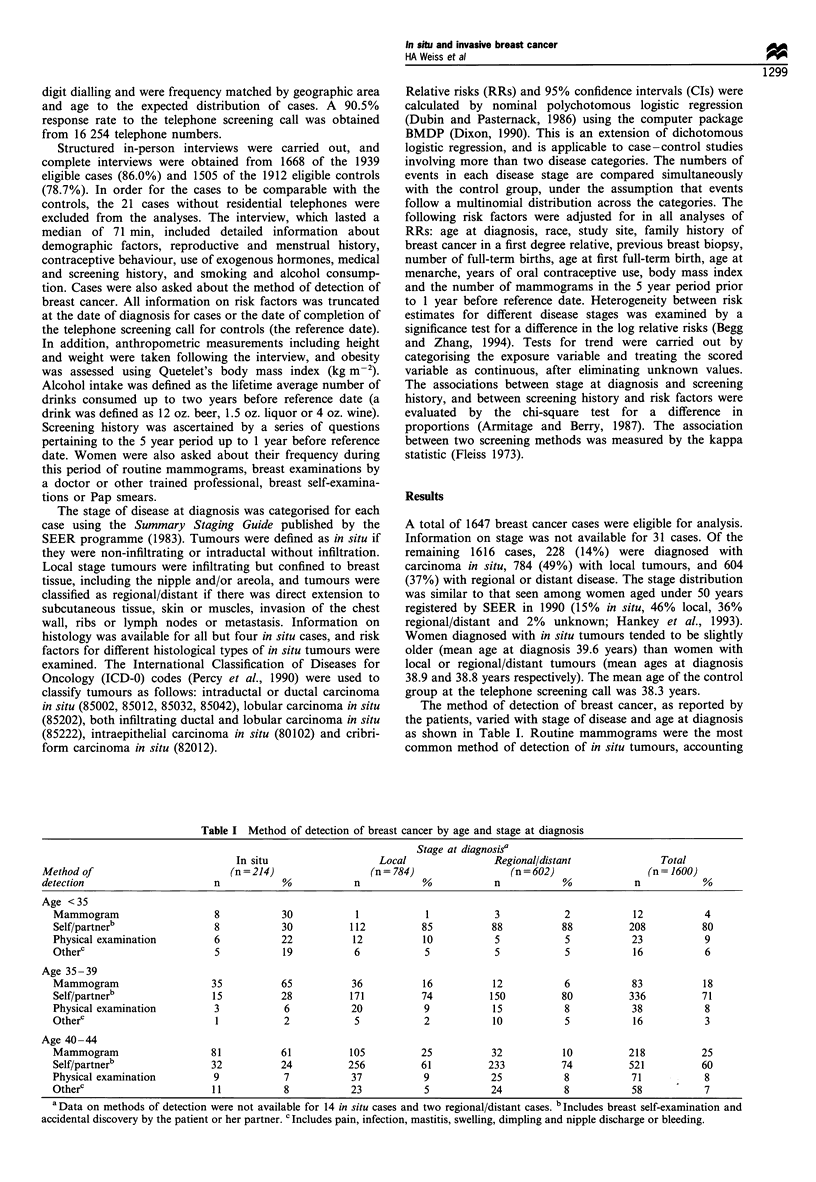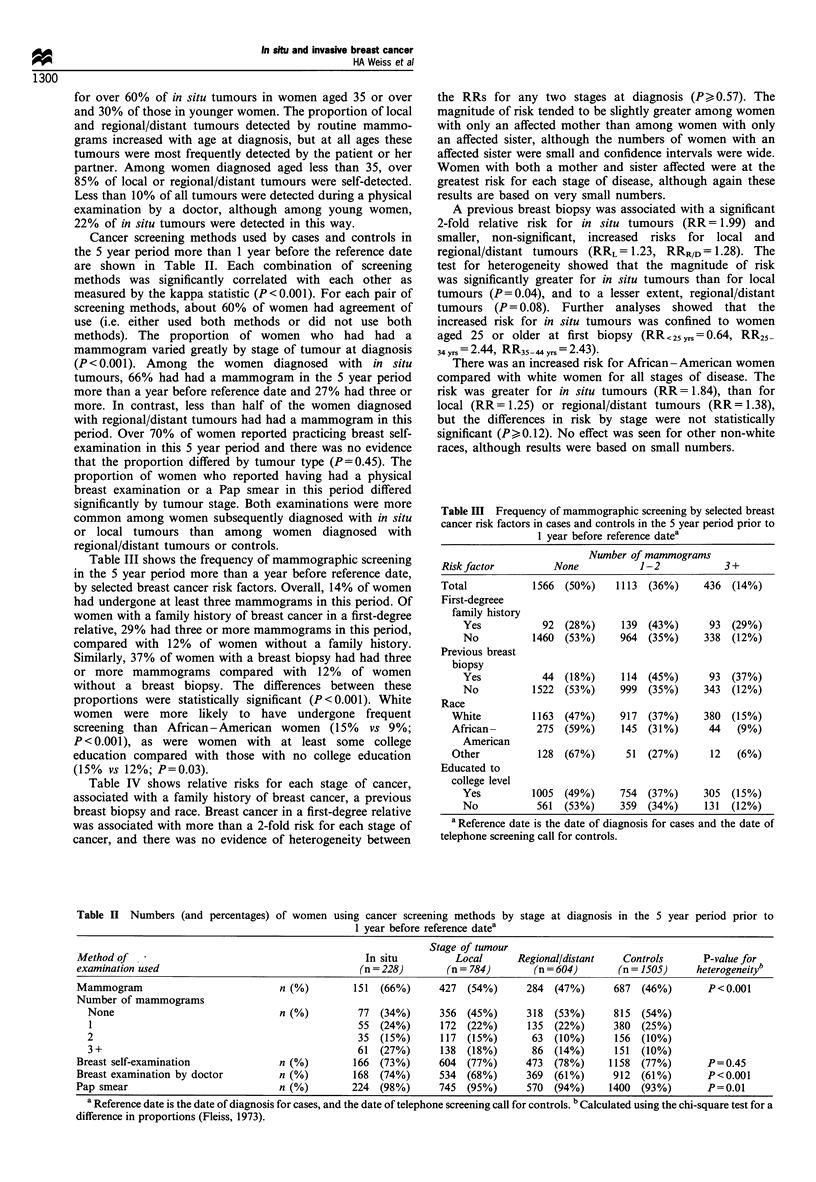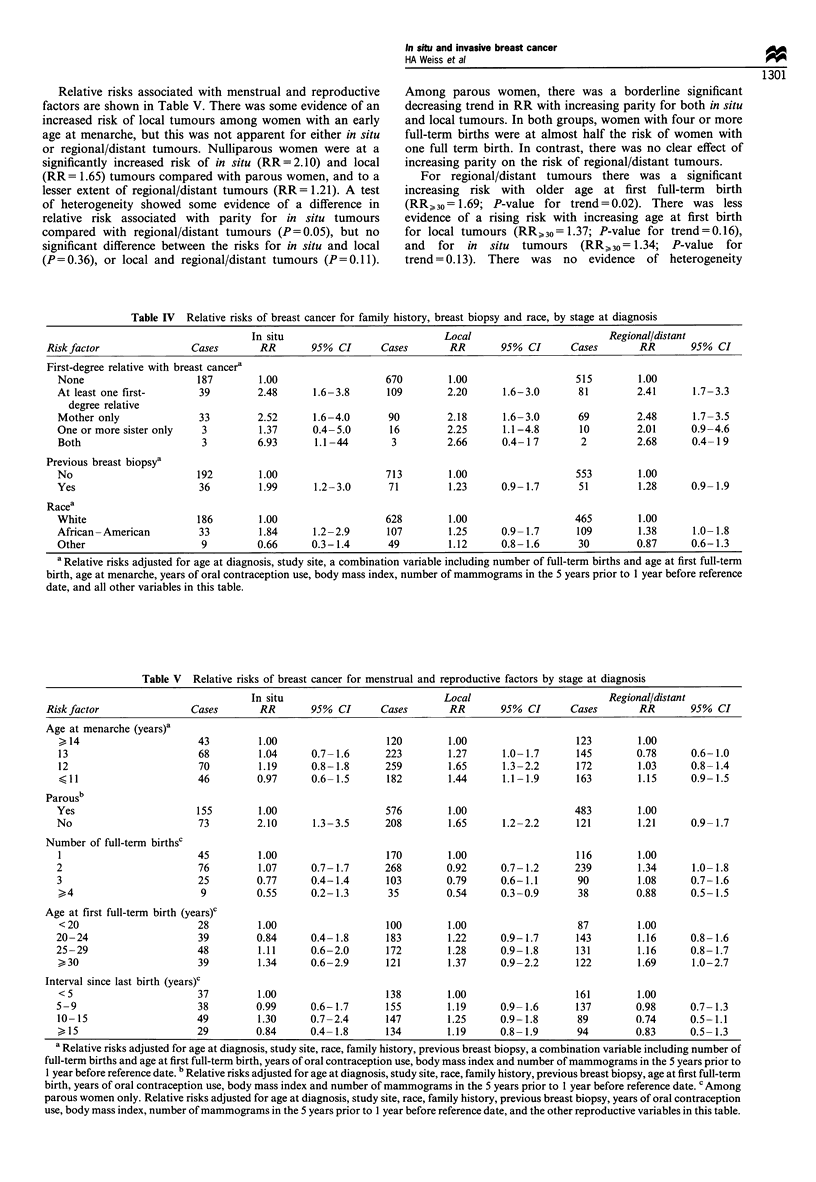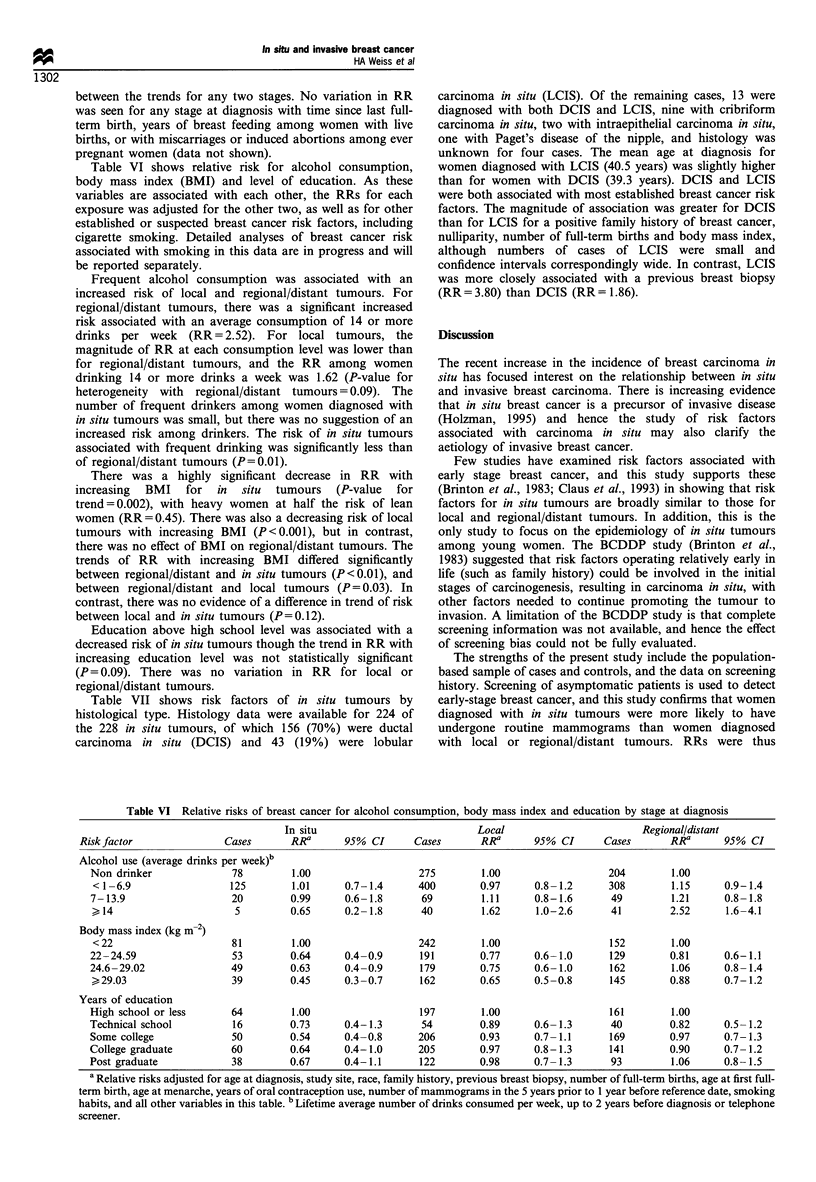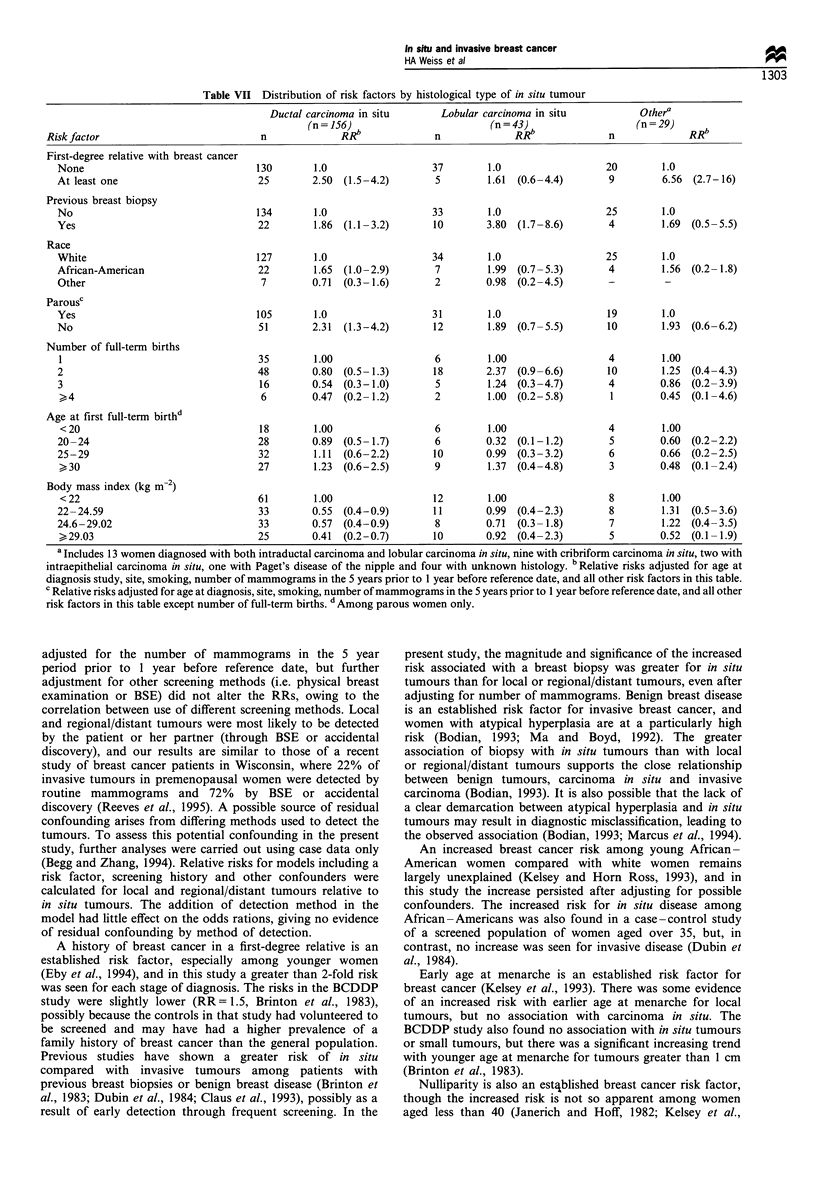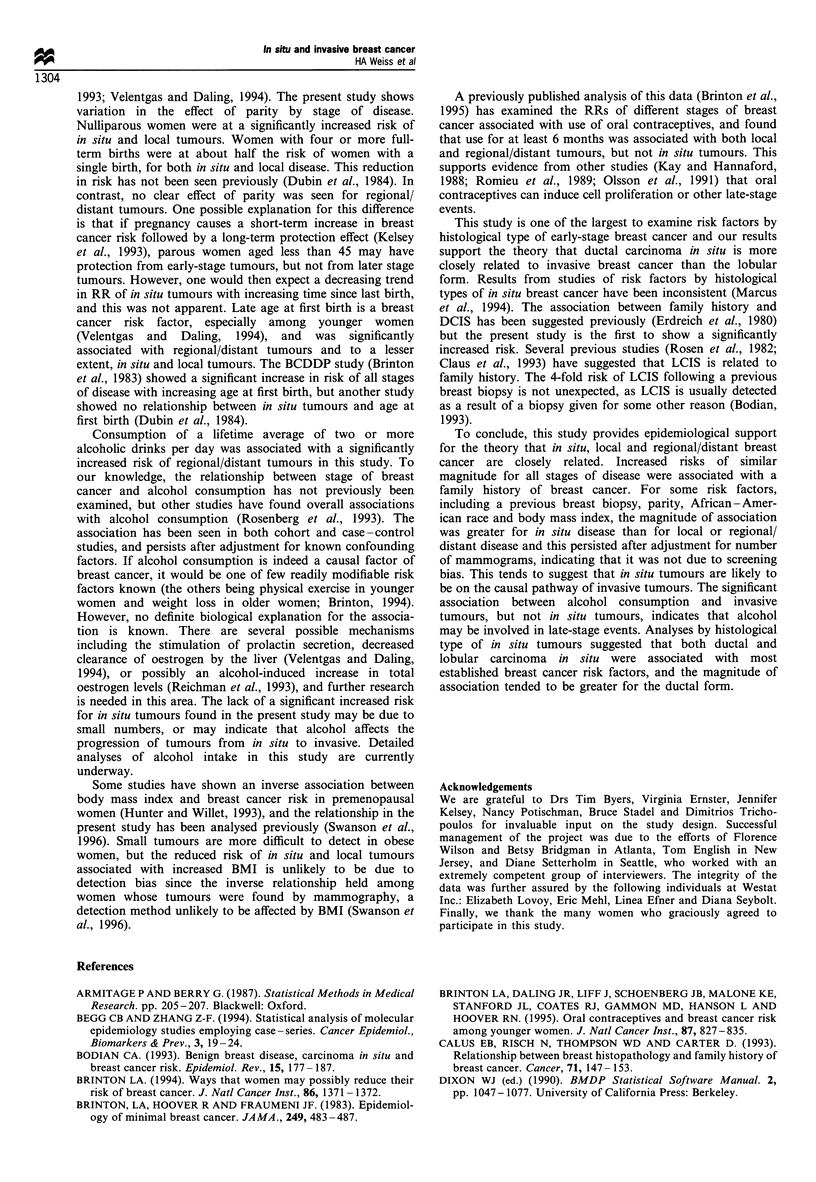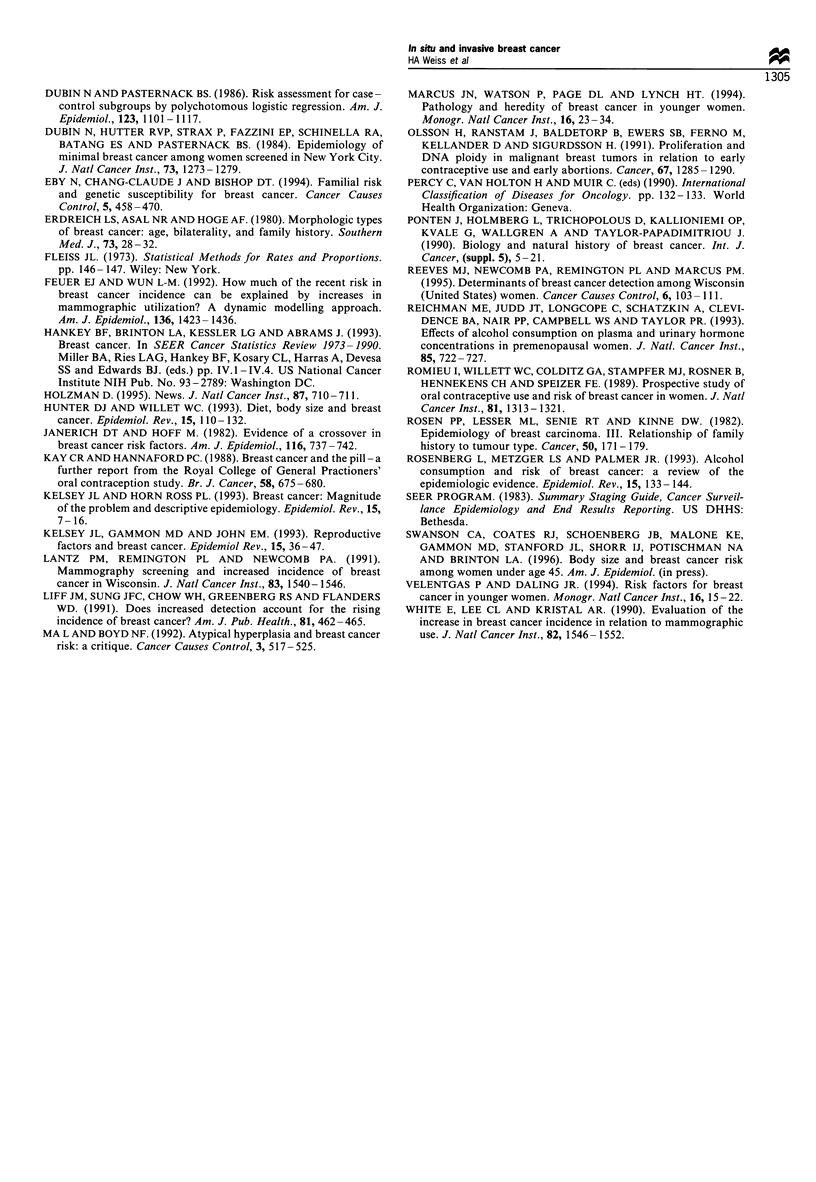# Epidemiology of in situ and invasive breast cancer in women aged under 45.

**DOI:** 10.1038/bjc.1996.248

**Published:** 1996-05

**Authors:** H. A. Weiss, L. A. Brinton, D. Brogan, R. J. Coates, M. D. Gammon, K. E. Malone, J. B. Schoenberg, C. A. Swanson

**Affiliations:** Environmental Epidemiology Branch, National Cancer Institute, Bethesda, MD 20892-7374, USA.

## Abstract

The incidence of in situ breast cancer in the USA has increased rapidly in recent years, even among young women. A population-based case-control study of 1616 breast cancer cases aged under 45 in the USA was used to examine risk factors for in situ, local and regional/distant tumours. Almost 60% of in situ tumours were detected by routine mammograms compared with 18% of local tumours and 8% of regional/distant tumours. After adjustment for screening history and established risk factors, family history of breast cancer in a first-degree relative and African-American race were associated with an increased risk of all stages of breast cancer. The associations with nulliparity, a previous breast biopsy and body mass index were significantly stronger for in situ tumours than for local or regional/distant disease. Alcohol consumption was associated with an increasing trend in risk of regional/distant tumours but not of earlier stage tumours, indicating that alcohol may be involved in late-stage events. Analyses by histological type of in situ tumours suggested that both ductal and lobular carcinoma in situ were associated with most established breast cancer risk factors, and the magnitude of association tended to be greater for the ductal form.


British Journal of Cancer (1996) 73, 1298-1305
© 1996 Stockton Press All rights reserved 0007-0920/96 $12.00

### Epidemiology of *in situ* and invasive breast cancer in women aged under 45

HA Weiss[1], LA Brinton[1], D Brogan[2], RJ Coates[3], MD Gammon[4], KE Malone[5], JB Schoenberg[6] and CA Swanson[1]

[1]*Environmental Epidemiology Branch, National Cancer Institute, Bethesda, MD 20892-7374; Departments of* [2]*Biostatistics and* [3]*Epidemiology, Rollins School of Public Health, Emory University, Atlanta, GA 30322;* [4]*Division of Epidemiology, Columbia University School of Public Health, New York, NY 10032;* [5]*Fred Hutchinson Cancer Research Center, Seattle, WA 98104;* [6]*Special Epidemiology Program, New Jersey State Department of Health, Trenton, NJ 08625, USA.*

**Summary** The incidence of *in situ* breast cancer in the USA has increased rapidly in recent years, even among young women. A population-based case–control study of 1616 breast cancer cases aged under 45 in the USA was used to examine risk factors for *in situ*, local and regional/distant tumours. Almost 60% of *in situ* tumours were detected by routine mammograms compared with 18% of local tumours and 8% of regional/distant tumours. After adjustment for screening history and established risk factors, family history of breast cancer in a first-degree relative and African–American race were associated with an increased risk of all stages of breast cancer. The associations with nulliparity, a previous breast biopsy and body mass index were significantly stronger for *in situ* tumours than for local or regional/distant disease. Alcohol consumption was associated with an increasing trend in risk of regional/distant tumours but not of earlier stage tumours, indicating that alcohol may be involved in late-stage events. Analyses by histological type of *in situ* tumours suggested that both ductal and lobular carcinoma *in situ* were associated with most established breast cancer risk factors, and the magnitude of association tended to be greater for the ductal form.

**Keywords:** breast cancer; carcinoma *in situ*; invasive breast cancer; epidemiology; premenopausal

The incidence of *in situ* carcinoma of the breast among women in the USA has increased about 4-fold since 1973, in contrast to only a slight increase in invasive breast cancer incidence (Hankey *et al.*, 1993). As a result, *in situ* tumours accounted for about 12% of diagnosed breast cancers in 1990, compared with less than 5% in the period 1973–80. The increased use of mammographic screening during these years explained most of the increase among older women (Lantz *et al.*, 1991; Liff *et al.*, 1991; Feuer and Wun, 1992). It is less likely that the 3-fold increase in incidence of *in situ* tumours that occurred among women aged less than 50 is caused by screening, owing to the low prevalence of screening among women in this age group (White *et al.*, 1990; Lantz *et al.*, 1991).

There are two main types of *in situ* breast carcinoma, the ductal and lobular forms, and their relationship with invasive breast cancer is not clearly understood. Ductal carcinoma *in situ* (DCIS) can be detected by mammography and is thought to represent a transitional stage in the development of an invasive tumour, with over 25–50% of tumours progressing to invasion, usually in the same breast (Ponten *et al.*, 1990; Bodian, 1993). In contrast, lobular carcinoma *in situ* is not clinically detectable by mammography and is usually an incidental finding during a biopsy. LCIS is probably a marker of high risk of subsequent invasive cancer in either breast, rather than a transitional stage in invasive malignancy (Ponten *et al.*, 1990) and the risk of developing invasive breast cancer following biopsy-treated LCIS is approximately 8% in both ipsilateral and contralateral breasts (Bodian, 1993). Evidence of the association between *in situ* and invasive disease (i.e. local or regional/distant tumours) was strengthened recently by research showing that the tumour-suppressor gene on chromosome 11 is mutated or missing in both invasive and *in situ* breast cancer (Holzman, 1995). The rapid increase in incidence of *in situ* tumours has prompted recent epidemiological studies to include *in situ* tumours as well as invasive tumours in analyses, but few studies have examined differences in risk factors by stage of disease. A follow-up study carried out within a nationwide screening programme [the Breast Cancer Demonstration Detection Project (BCDDP), Brinton *et al.*, 1983] found a number of shared risk factors for *in situ* and invasive tumours, including a family history of breast cancer, previous breast biopsy and late age at first livebirth. However, this study was limited by lack of information on complete screening history. Results from another study (Dubin *et al.*, 1984) showed no evidence that *in situ* tumours were associated with family history of breast cancer or a previous breast biopsy, although there was a significant association with a breast lump or cyst, and for African–American women compared with white women.

The present case–control study is the largest study of women aged under 45 to compare risk factors for *in situ*, local and regional/distant breast cancer. In addition, risk factors for histological types of *in situ* tumours have been examined. The role of screening bias is especially important in studies of non-invasive tumours, as screening procedures such as frequent mammograms are likely to detect tumours at an early stage. In this study, detailed screening information was collected at the time of interview for cases and controls, allowing the effect of screening on stage at diagnosis to be evaluated.

### Materials and methods

This population-based case–control study was conducted in three different geographic areas of the USA covered by cancer registries–Atlanta, Seattle/Puget Sound and five counties in central New Jersey. Study details have been published elsewhere (Brinton *et al.*, 1995). Briefly, the present analyses consist of women aged 20–44 years who were newly diagnosed with breast cancer during the period 1 May 1990 to 31 December 1992. Cases were identified through rapid ascertainment systems, and histological information on stage at diagnosis was obtained from the Cancer Surveillance Epidemiology and End Results (SEER) programme for cases from Atlanta and Seattle, and from hospital records for cases from New Jersey. Controls were chosen through random

Correspondence: HA Weiss, Environmental Epidemiology Branch, National Cancer Institute, Executive Plaza North, Room 443, 6130 Exec. Blvd, Bethesda, MD 20892-7374, USA.
Received 9 October 1995; revised 18 December 1995; accepted 18 December 1995

digit dialling and were frequency matched by geographic area and age to the expected distribution of cases. A 90.5% response rate to the telephone screening call was obtained from 16 254 telephone numbers.

Structured in-person interviews were carried out, and complete interviews were obtained from 1668 of the 1939 eligible cases (86.0%) and 1505 of the 1912 eligible controls (78.7%). In order for the cases to be comparable with the controls, the 21 cases without residential telephones were excluded from the analyses. The interview, which lasted a median of 71 min, included detailed information about demographic factors, reproductive and menstrual history, contraceptive behaviour, use of exogenous hormones, medical and screening history, and smoking and alcohol consumption. Cases were also asked about the method of detection of breast cancer. All information on risk factors was truncated at the date of diagnosis for cases or the date of completion of the telephone screening call for controls (the reference date). In addition, anthropometric measurements including height and weight were taken following the interview, and obesity was assessed using Quetelet's body mass index (kg $m^{-2}$). Alcohol intake was defined as the lifetime average number of drinks consumed up to two years before reference date (a drink was defined as 12 oz. beer, 1.5 oz. liquor or 4 oz. wine). Screening history was ascertained by a series of questions pertaining to the 5 year period up to 1 year before reference date. Women were also asked about their frequency during this period of routine mammograms, breast examinations by a doctor or other trained professional, breast self-examinations or Pap smears.

The stage of disease at diagnosis was categorised for each case using the *Summary Staging Guide* published by the SEER programme (1983). Tumours were defined as *in situ* if they were non-infiltrating or intraductal without infiltration. Local stage tumours were infiltrating but confined to breast tissue, including the nipple and/or areola, and tumours were classified as regional/distant if there was direct extension to subcutaneous tissue, skin or muscles, invasion of the chest wall, ribs or lymph nodes or metastasis. Information on histology was available for all but four *in situ* cases, and risk factors for different histological types of *in situ* tumours were examined. The International Classification of Diseases for Oncology (ICD-0) codes (Percy *et al.*, 1990) were used to classify tumours as follows: intraductal or ductal carcinoma *in situ* (85002, 85012, 85032, 85042), lobular carcinoma *in situ* (85202), both infiltrating ductal and lobular carcinoma *in situ* (85222), intraepithelial carcinoma *in situ* (80102) and cribriform carcinoma *in situ* (82012).

Relative risks (RRs) and 95% confidence intervals (CIs) were calculated by nominal polychotomous logistic regression (Dubin and Pasternack, 1986) using the computer package BMDP (Dixon, 1990). This is an extension of dichotomous logistic regression, and is applicable to case-control studies involving more than two disease categories. The numbers of events in each disease stage are compared simultaneously with the control group, under the assumption that events follow a multinomial distribution across the categories. The following risk factors were adjusted for in all analyses of RRs: age at diagnosis, race, study site, family history of breast cancer in a first degree relative, previous breast biopsy, number of full-term births, age at first full-term birth, age at menarche, years of oral contraceptive use, body mass index and the number of mammograms in the 5 year period prior to 1 year before reference date. Heterogeneity between risk estimates for different disease stages was examined by a significance test for a difference in the log relative risks (Begg and Zhang, 1994). Tests for trend were carried out by categorising the exposure variable and treating the scored variable as continuous, after eliminating unknown values. The associations between stage at diagnosis and screening history, and between screening history and risk factors were evaluated by the chi-square test for a difference in proportions (Armitage and Berry, 1987). The association between two screening methods was measured by the kappa statistic (Fleiss 1973).

### Results

A total of 1647 breast cancer cases were eligible for analysis. Information on stage was not available for 31 cases. Of the remaining 1616 cases, 228 (14%) were diagnosed with carcinoma *in situ*, 784 (49%) with local tumours, and 604 (37%) with regional or distant disease. The stage distribution was similar to that seen among women aged under 50 years registered by SEER in 1990 (15% *in situ*, 46% local, 36% regional/distant and 2% unknown; Hankey *et al.*, 1993). Women diagnosed with *in situ* tumours tended to be slightly older (mean age at diagnosis 39.6 years) than women with local or regional/distant tumours (mean ages at diagnosis 38.9 and 38.8 years respectively). The mean age of the control group at the telephone screening call was 38.3 years.

The method of detection of breast cancer, as reported by the patients, varied with stage of disease and age at diagnosis as shown in Table I. Routine mammograms were the most common method of detection of *in situ* tumours, accounting

**Table I** Method of detection of breast cancer by age and stage at diagnosis

| Method of detection | In situ (n=214) n | % | *Local* (n=784) n | % | *Regional/distant* (n=602) n | % | *Total* (n=1600) n | % |
|---|---|---|---|---|---|---|---|---|
| Age <35 | | | | | | | | |
| Mammogram | 8 | 30 | 1 | 1 | 3 | 2 | 12 | 4 |
| Self/partner[b] | 8 | 30 | 112 | 85 | 88 | 88 | 208 | 80 |
| Physical examination | 6 | 22 | 12 | 10 | 5 | 5 | 23 | 9 |
| Other[c] | 5 | 19 | 6 | 5 | 5 | 5 | 16 | 6 |
| Age 35-39 | | | | | | | | |
| Mammogram | 35 | 65 | 36 | 16 | 12 | 6 | 83 | 18 |
| Self/partner[b] | 15 | 28 | 171 | 74 | 150 | 80 | 336 | 71 |
| Physical examination | 3 | 6 | 20 | 9 | 15 | 8 | 38 | 8 |
| Other[c] | 1 | 2 | 5 | 2 | 10 | 5 | 16 | 3 |
| Age 40-44 | | | | | | | | |
| Mammogram | 81 | 61 | 105 | 25 | 32 | 10 | 218 | 25 |
| Self/partner[b] | 32 | 24 | 256 | 61 | 233 | 74 | 521 | 60 |
| Physical examination | 9 | 7 | 37 | 9 | 25 | 8 | 71 | 8 |
| Other[c] | 11 | 8 | 23 | 5 | 24 | 8 | 58 | 7 |

*Stage at diagnosis*[a]

[a] Data on methods of detection were not available for 14 *in situ* cases and two regional/distant cases. [b] Includes breast self-examination and accidental discovery by the patient or her partner. [c] Includes pain, infection, mastitis, swelling, dimpling and nipple discharge or bleeding.

for over 60% of *in situ* tumours in women aged 35 or over and 30% of those in younger women. The proportion of local and regional/distant tumours detected by routine mammograms increased with age at diagnosis, but at all ages these tumours were most frequently detected by the patient or her partner. Among women diagnosed aged less than 35, over 85% of local or regional/distant tumours were self-detected. Less than 10% of all tumours were detected during a physical examination by a doctor, although among young women, 22% of *in situ* tumours were detected in this way.

Cancer screening methods used by cases and controls in the 5 year period more than 1 year before the reference date are shown in Table II. Each combination of screening methods was significantly correlated with each other as measured by the kappa statistic ($P<0.001$). For each pair of screening methods, about 60% of women had agreement of use (i.e. either used both methods or did not use both methods). The proportion of women who had had a mammogram varied greatly by stage of tumour at diagnosis ($P<0.001$). Among the women diagnosed with *in situ* tumours, 66% had had a mammogram in the 5 year period more than a year before reference date and 27% had three or more. In contrast, less than half of the women diagnosed with regional/distant tumours had had a mammogram in this period. Over 70% of women reported practicing breast self-examination in this 5 year period and there was no evidence that the proportion differed by tumour type ($P=0.45$). The proportion of women who reported having had a physical breast examination or a Pap smear in this period differed significantly by tumour stage. Both examinations were more common among women subsequently diagnosed with *in situ* or local tumours than among women diagnosed with regional/distant tumours or controls.

Table III shows the frequency of mammographic screening in the 5 year period more than a year before reference date, by selected breast cancer risk factors. Overall, 14% of women had undergone at least three mammograms in this period. Of women with a family history of breast cancer in a first-degree relative, 29% had three or more mammograms in this period, compared with 12% of women without a family history. Similarly, 37% of women with a breast biopsy had had three or more mammograms compared with 12% of women without a breast biopsy. The differences between these proportions were statistically significant ($P<0.001$). White women were more likely to have undergone frequent screening than African-American women (15% *vs* 9%; $P<0.001$), as were women with at least some college education compared with those with no college education (15% *vs* 12%; $P=0.03$).

Table IV shows relative risks for each stage of cancer, associated with a family history of breast cancer, a previous breast biopsy and race. Breast cancer in a first-degree relative was associated with more than a 2-fold risk for each stage of cancer, and there was no evidence of heterogeneity between the RRs for any two stages at diagnosis ($P \geqslant 0.57$). The magnitude of risk tended to be slightly greater among women with only an affected mother than among women with only an affected sister, although the numbers of women with an affected sister were small and confidence intervals were wide. Women with both a mother and sister affected were at the greatest risk for each stage of disease, although again these results are based on very small numbers.

A previous breast biopsy was associated with a significant 2-fold relative risk for *in situ* tumours (RR = 1.99) and smaller, non-significant, increased risks for local and regional/distant tumours ($RR_L=1.23$, $RR_{R/D}=1.28$). The test for heterogeneity showed that the magnitude of risk was significantly greater for *in situ* tumours than for local tumours ($P=0.04$), and to a lesser extent, regional/distant tumours ($P=0.08$). Further analyses showed that the increased risk for *in situ* tumours was confined to women aged 25 or older at first biopsy ($RR_{<25\ yrs}=0.64$, $RR_{25-34\ yrs}=2.44$, $RR_{35-44\ yrs}=2.43$).

There was an increased risk for African-American women compared with white women for all stages of disease. The risk was greater for *in situ* tumours (RR = 1.84), than for local (RR = 1.25) or regional/distant tumours (RR = 1.38), but the differences in risk by stage were not statistically significant ($P \geqslant 0.12$). No effect was seen for other non-white races, although results were based on small numbers.

**Table III** Frequency of mammographic screening by selected breast cancer risk factors in cases and controls in the 5 year period prior to 1 year before reference date[a]

| *Risk factor* | *Number of mammograms* None | | 1–2 | | 3+ | |
|---|---|---|---|---|---|---|
| Total | 1566 | (50%) | 1113 | (36%) | 436 | (14%) |
| First-degreee family history | | | | | | |
| Yes | 92 | (28%) | 139 | (43%) | 93 | (29%) |
| No | 1460 | (53%) | 964 | (35%) | 338 | (12%) |
| Previous breast biopsy | | | | | | |
| Yes | 44 | (18%) | 114 | (45%) | 93 | (37%) |
| No | 1522 | (53%) | 999 | (35%) | 343 | (12%) |
| Race | | | | | | |
| White | 1163 | (47%) | 917 | (37%) | 380 | (15%) |
| African-American | 275 | (59%) | 145 | (31%) | 44 | (9%) |
| Other | 128 | (67%) | 51 | (27%) | 12 | (6%) |
| Educated to college level | | | | | | |
| Yes | 1005 | (49%) | 754 | (37%) | 305 | (15%) |
| No | 561 | (53%) | 359 | (34%) | 131 | (12%) |

[a] Reference date is the date of diagnosis for cases and the date of telephone screening call for controls.

**Table II** Numbers (and percentages) of women using cancer screening methods by stage at diagnosis in the 5 year period prior to 1 year before reference date[a]

| *Method of examination used* | | *Stage of tumour* In situ (n = 228) | | Local (n = 784) | | Regional/distant (n = 604) | | Controls (n = 1505) | | P-*value for heterogeneity*[b] |
|---|---|---|---|---|---|---|---|---|---|---|
| Mammogram | *n* (%) | 151 | (66%) | 427 | (54%) | 284 | (47%) | 687 | (46%) | $P<0.001$ |
| Number of mammograms | | | | | | | | | | |
| None | *n* (%) | 77 | (34%) | 356 | (45%) | 318 | (53%) | 815 | (54%) | |
| 1 | | 55 | (24%) | 172 | (22%) | 135 | (22%) | 380 | (25%) | |
| 2 | | 35 | (15%) | 117 | (15%) | 63 | (10%) | 156 | (10%) | |
| 3+ | | 61 | (27%) | 138 | (18%) | 86 | (14%) | 151 | (10%) | |
| Breast self-examination | *n* (%) | 166 | (73%) | 604 | (77%) | 473 | (78%) | 1158 | (77%) | $P=0.45$ |
| Breast examination by doctor | *n* (%) | 168 | (74%) | 534 | (68%) | 369 | (61%) | 912 | (61%) | $P<0.001$ |
| Pap smear | *n* (%) | 224 | (98%) | 745 | (95%) | 570 | (94%) | 1400 | (93%) | $P=0.01$ |

[a] Reference date is the date of diagnosis for cases, and the date of telephone screening call for controls. [b] Calculated using the chi-square test for a difference in proportions (Fleiss, 1973).

Relative risks associated with menstrual and reproductive factors are shown in Table V. There was some evidence of an increased risk of local tumours among women with an early age at menarche, but this was not apparent for either *in situ* or regional/distant tumours. Nulliparous women were at a significantly increased risk of *in situ* (RR = 2.10) and local (RR = 1.65) tumours compared with parous women, and to a lesser extent of regional/distant tumours (RR = 1.21). A test of heterogeneity showed some evidence of a difference in relative risk associated with parity for *in situ* tumours compared with regional/distant tumours ($P = 0.05$), but no significant difference between the risks for *in situ* and local ($P = 0.36$), or local and regional/distant tumours ($P = 0.11$).

Among parous women, there was a borderline significant decreasing trend in RR with increasing parity for both *in situ* and local tumours. In both groups, women with four or more full-term births were at almost half the risk of women with one full term birth. In contrast, there was no clear effect of increasing parity on the risk of regional/distant tumours.

For regional/distant tumours there was a significant increasing risk with older age at first full-term birth ($RR_{\geqslant 30} = 1.69$; $P$-value for trend = 0.02). There was less evidence of a rising risk with increasing age at first birth for local tumours ($RR_{\geqslant 30} = 1.37$; $P$-value for trend = 0.16), and for *in situ* tumours ($RR_{\geqslant 30} = 1.34$; $P$-value for trend = 0.13). There was no evidence of heterogeneity

**Table IV** Relative risks of breast cancer for family history, breast biopsy and race, by stage at diagnosis

| | | In situ | | | Local | | | Regional/distant | |
|---|---|---|---|---|---|---|---|---|---|
| *Risk factor* | *Cases* | *RR* | *95% CI* | *Cases* | *RR* | *95% CI* | *Cases* | *RR* | *95% CI* |
| First-degree relative with breast cancer[a] | | | | | | | | | |
| None | 187 | 1.00 | | 670 | 1.00 | | 515 | 1.00 | |
| At least one first-degree relative | 39 | 2.48 | 1.6–3.8 | 109 | 2.20 | 1.6–3.0 | 81 | 2.41 | 1.7–3.3 |
| Mother only | 33 | 2.52 | 1.6–4.0 | 90 | 2.18 | 1.6–3.0 | 69 | 2.48 | 1.7–3.5 |
| One or more sister only | 3 | 1.37 | 0.4–5.0 | 16 | 2.25 | 1.1–4.8 | 10 | 2.01 | 0.9–4.6 |
| Both | 3 | 6.93 | 1.1–44 | 3 | 2.66 | 0.4–17 | 2 | 2.68 | 0.4–19 |
| Previous breast biopsy[a] | | | | | | | | | |
| No | 192 | 1.00 | | 713 | 1.00 | | 553 | 1.00 | |
| Yes | 36 | 1.99 | 1.2–3.0 | 71 | 1.23 | 0.9–1.7 | 51 | 1.28 | 0.9–1.9 |
| Race[a] | | | | | | | | | |
| White | 186 | 1.00 | | 628 | 1.00 | | 465 | 1.00 | |
| African–American | 33 | 1.84 | 1.2–2.9 | 107 | 1.25 | 0.9–1.7 | 109 | 1.38 | 1.0–1.8 |
| Other | 9 | 0.66 | 0.3–1.4 | 49 | 1.12 | 0.8–1.6 | 30 | 0.87 | 0.6–1.3 |

[a] Relative risks adjusted for age at diagnosis, study site, a combination variable including number of full-term births and age at first full-term birth, age at menarche, years of oral contraception use, body mass index, number of mammograms in the 5 years prior to 1 year before reference date, and all other variables in this table.

**Table V** Relative risks of breast cancer for menstrual and reproductive factors by stage at diagnosis

| | | In situ | | | Local | | | Regional/distant | |
|---|---|---|---|---|---|---|---|---|---|
| *Risk factor* | *Cases* | *RR* | *95% CI* | *Cases* | *RR* | *95% CI* | *Cases* | *RR* | *95% CI* |
| Age at menarche (years)[a] | | | | | | | | | |
| ⩾14 | 43 | 1.00 | | 120 | 1.00 | | 123 | 1.00 | |
| 13 | 68 | 1.04 | 0.7–1.6 | 223 | 1.27 | 1.0–1.7 | 145 | 0.78 | 0.6–1.0 |
| 12 | 70 | 1.19 | 0.8–1.8 | 259 | 1.65 | 1.3–2.2 | 172 | 1.03 | 0.8–1.4 |
| ⩽11 | 46 | 0.97 | 0.6–1.5 | 182 | 1.44 | 1.1–1.9 | 163 | 1.15 | 0.9–1.5 |
| Parous[b] | | | | | | | | | |
| Yes | 155 | 1.00 | | 576 | 1.00 | | 483 | 1.00 | |
| No | 73 | 2.10 | 1.3–3.5 | 208 | 1.65 | 1.2–2.2 | 121 | 1.21 | 0.9–1.7 |
| Number of full-term births[c] | | | | | | | | | |
| 1 | 45 | 1.00 | | 170 | 1.00 | | 116 | 1.00 | |
| 2 | 76 | 1.07 | 0.7–1.7 | 268 | 0.92 | 0.7–1.2 | 239 | 1.34 | 1.0–1.8 |
| 3 | 25 | 0.77 | 0.4–1.4 | 103 | 0.79 | 0.6–1.1 | 90 | 1.08 | 0.7–1.6 |
| ⩾4 | 9 | 0.55 | 0.2–1.3 | 35 | 0.54 | 0.3–0.9 | 38 | 0.88 | 0.5–1.5 |
| Age at first full-term birth (years)[c] | | | | | | | | | |
| <20 | 28 | 1.00 | | 100 | 1.00 | | 87 | 1.00 | |
| 20–24 | 39 | 0.84 | 0.4–1.8 | 183 | 1.22 | 0.9–1.7 | 143 | 1.16 | 0.8–1.6 |
| 25–29 | 48 | 1.11 | 0.6–2.0 | 172 | 1.28 | 0.9–1.8 | 131 | 1.16 | 0.8–1.7 |
| ⩾30 | 39 | 1.34 | 0.6–2.9 | 121 | 1.37 | 0.9–2.2 | 122 | 1.69 | 1.0–2.7 |
| Interval since last birth (years)[c] | | | | | | | | | |
| <5 | 37 | 1.00 | | 138 | 1.00 | | 161 | 1.00 | |
| 5–9 | 38 | 0.99 | 0.6–1.7 | 155 | 1.19 | 0.9–1.6 | 137 | 0.98 | 0.7–1.3 |
| 10–15 | 49 | 1.30 | 0.7–2.4 | 147 | 1.25 | 0.9–1.8 | 89 | 0.74 | 0.5–1.1 |
| ⩾15 | 29 | 0.84 | 0.4–1.8 | 134 | 1.19 | 0.8–1.9 | 94 | 0.83 | 0.5–1.3 |

[a] Relative risks adjusted for age at diagnosis, study site, race, family history, previous breast biopsy, a combination variable including number of full-term births and age at first full-term birth, years of oral contraception use, body mass index and number of mammograms in the 5 years prior to 1 year before reference date. [b] Relative risks adjusted for age at diagnosis, study site, race, family history, previous breast biopsy, age at first full-term birth, years of oral contraception use, body mass index and number of mammograms in the 5 years prior to 1 year before reference date. [c] Among parous women only. Relative risks adjusted for age at diagnosis, study site, race, family history, previous breast biopsy, years of oral contraception use, body mass index, number of mammograms in the 5 years prior to 1 year before reference date, and the other reproductive variables in this table.

between the trends for any two stages. No variation in RR was seen for any stage at diagnosis with time since last full-term birth, years of breast feeding among women with live births, or with miscarriages or induced abortions among ever pregnant women (data not shown).

Table VI shows relative risk for alcohol consumption, body mass index (BMI) and level of education. As these variables are associated with each other, the RRs for each exposure was adjusted for the other two, as well as for other established or suspected breast cancer risk factors, including cigarette smoking. Detailed analyses of breast cancer risk associated with smoking in this data are in progress and will be reported separately.

Frequent alcohol consumption was associated with an increased risk of local and regional/distant tumours. For regional/distant tumours, there was a significant increased risk associated with an average consumption of 14 or more drinks per week (RR=2.52). For local tumours, the magnitude of RR at each consumption level was lower than for regional/distant tumours, and the RR among women drinking 14 or more drinks a week was 1.62 ($P$-value for heterogeneity with regional/distant tumours=0.09). The number of frequent drinkers among women diagnosed with *in situ* tumours was small, but there was no suggestion of an increased risk among drinkers. The risk of *in situ* tumours associated with frequent drinking was significantly less than of regional/distant tumours ($P=0.01$).

There was a highly significant decrease in RR with increasing BMI for *in situ* tumours ($P$-value for trend=0.002), with heavy women at half the risk of lean women (RR=0.45). There was also a decreasing risk of local tumours with increasing BMI ($P<0.001$), but in contrast, there was no effect of BMI on regional/distant tumours. The trends of RR with increasing BMI differed significantly between regional/distant and *in situ* tumours ($P<0.01$), and between regional/distant and local tumours ($P=0.03$). In contrast, there was no evidence of a difference in trend of risk between local and *in situ* tumours ($P=0.12$).

Education above high school level was associated with a decreased risk of *in situ* tumours though the trend in RR with increasing education level was not statistically significant ($P=0.09$). There was no variation in RR for local or regional/distant tumours.

Table VII shows risk factors of *in situ* tumours by histological type. Histology data were available for 224 of the 228 *in situ* tumours, of which 156 (70%) were ductal carcinoma *in situ* (DCIS) and 43 (19%) were lobular carcinoma *in situ* (LCIS). Of the remaining cases, 13 were diagnosed with both DCIS and LCIS, nine with cribriform carcinoma *in situ*, two with intraepithelial carcinoma *in situ*, one with Paget's disease of the nipple, and histology was unknown for four cases. The mean age at diagnosis for women diagnosed with LCIS (40.5 years) was slightly higher than for women with DCIS (39.3 years). DCIS and LCIS were both associated with most established breast cancer risk factors. The magnitude of association was greater for DCIS than for LCIS for a positive family history of breast cancer, nulliparity, number of full-term births and body mass index, although numbers of cases of LCIS were small and confidence intervals correspondingly wide. In contrast, LCIS was more closely associated with a previous breast biopsy (RR=3.80) than DCIS (RR=1.86).

### Discussion

The recent increase in the incidence of breast carcinoma *in situ* has focused interest on the relationship between *in situ* and invasive breast carcinoma. There is increasing evidence that *in situ* breast cancer is a precursor of invasive disease (Holzman, 1995) and hence the study of risk factors associated with carcinoma *in situ* may also clarify the aetiology of invasive breast cancer.

Few studies have examined risk factors associated with early stage breast cancer, and this study supports these (Brinton *et al*., 1983; Claus *et al*., 1993) in showing that risk factors for *in situ* tumours are broadly similar to those for local and regional/distant tumours. In addition, this is the only study to focus on the epidemiology of *in situ* tumours among young women. The BCDDP study (Brinton *et al*., 1983) suggested that risk factors operating relatively early in life (such as family history) could be involved in the initial stages of carcinogenesis, resulting in carcinoma *in situ*, with other factors needed to continue promoting the tumour to invasion. A limitation of the BCDDP study is that complete screening information was not available, and hence the effect of screening bias could not be fully evaluated.

The strengths of the present study include the population-based sample of cases and controls, and the data on screening history. Screening of asymptomatic patients is used to detect early-stage breast cancer, and this study confirms that women diagnosed with *in situ* tumours were more likely to have undergone routine mammograms than women diagnosed with local or regional/distant tumours. RRs were thus

**Table VI** Relative risks of breast cancer for alcohol consumption, body mass index and education by stage at diagnosis

| *Risk factor* | *Cases* | In situ *RR*[a] | *95% CI* | *Cases* | *Local* *RR*[a] | *95% CI* | *Cases* | *Regional/distant* *RR*[a] | *95% CI* |
|---|---|---|---|---|---|---|---|---|---|
| Alcohol use (average drinks per week)[b] | | | | | | | | | |
| Non drinker | 78 | 1.00 | | 275 | 1.00 | | 204 | 1.00 | |
| <1–6.9 | 125 | 1.01 | 0.7–1.4 | 400 | 0.97 | 0.8–1.2 | 308 | 1.15 | 0.9–1.4 |
| 7–13.9 | 20 | 0.99 | 0.6–1.8 | 69 | 1.11 | 0.8–1.6 | 49 | 1.21 | 0.8–1.8 |
| ⩾14 | 5 | 0.65 | 0.2–1.8 | 40 | 1.62 | 1.0–2.6 | 41 | 2.52 | 1.6–4.1 |
| Body mass index (kg $m^{-2}$) | | | | | | | | | |
| <22 | 81 | 1.00 | | 242 | 1.00 | | 152 | 1.00 | |
| 22–24.59 | 53 | 0.64 | 0.4–0.9 | 191 | 0.77 | 0.6–1.0 | 129 | 0.81 | 0.6–1.1 |
| 24.6–29.02 | 49 | 0.63 | 0.4–0.9 | 179 | 0.75 | 0.6–1.0 | 162 | 1.06 | 0.8–1.4 |
| ⩾29.03 | 39 | 0.45 | 0.3–0.7 | 162 | 0.65 | 0.5–0.8 | 145 | 0.88 | 0.7–1.2 |
| Years of education | | | | | | | | | |
| High school or less | 64 | 1.00 | | 197 | 1.00 | | 161 | 1.00 | |
| Technical school | 16 | 0.73 | 0.4–1.3 | 54 | 0.89 | 0.6–1.3 | 40 | 0.82 | 0.5–1.2 |
| Some college | 50 | 0.54 | 0.4–0.8 | 206 | 0.93 | 0.7–1.1 | 169 | 0.97 | 0.7–1.3 |
| College graduate | 60 | 0.64 | 0.4–1.0 | 205 | 0.97 | 0.8–1.3 | 141 | 0.90 | 0.7–1.2 |
| Post graduate | 38 | 0.67 | 0.4–1.1 | 122 | 0.98 | 0.7–1.3 | 93 | 1.06 | 0.8–1.5 |

[a] Relative risks adjusted for age at diagnosis, study site, race, family history, previous breast biopsy, number of full-term births, age at first full-term birth, age at menarche, years of oral contraception use, number of mammograms in the 5 years prior to 1 year before reference date, smoking habits, and all other variables in this table. [b] Lifetime average number of drinks consumed per week, up to 2 years before diagnosis or telephone screener.

Table VII Distribution of risk factors by histological type of *in situ* tumour

| Risk factor | *Ductal carcinoma* in situ (n=156) n | RR[b] | | *Lobular carcinoma* in situ (n=43) n | RR[b] | | *Other*[a] (n=29) n | RR[b] | |
|---|---|---|---|---|---|---|---|---|---|
| First-degree relative with breast cancer | | | | | | | | | |
| None | 130 | 1.0 | | 37 | 1.0 | | 20 | 1.0 | |
| At least one | 25 | 2.50 | (1.5–4.2) | 5 | 1.61 | (0.6–4.4) | 9 | 6.56 | (2.7–16) |
| Previous breast biopsy | | | | | | | | | |
| No | 134 | 1.0 | | 33 | 1.0 | | 25 | 1.0 | |
| Yes | 22 | 1.86 | (1.1–3.2) | 10 | 3.80 | (1.7–8.6) | 4 | 1.69 | (0.5–5.5) |
| Race | | | | | | | | | |
| White | 127 | 1.0 | | 34 | 1.0 | | 25 | 1.0 | |
| African-American | 22 | 1.65 | (1.0–2.9) | 7 | 1.99 | (0.7–5.3) | 4 | 1.56 | (0.2–1.8) |
| Other | 7 | 0.71 | (0.3–1.6) | 2 | 0.98 | (0.2–4.5) | – | – | |
| Parous[c] | | | | | | | | | |
| Yes | 105 | 1.0 | | 31 | 1.0 | | 19 | 1.0 | |
| No | 51 | 2.31 | (1.3–4.2) | 12 | 1.89 | (0.7–5.5) | 10 | 1.93 | (0.6–6.2) |
| Number of full-term births | | | | | | | | | |
| 1 | 35 | 1.00 | | 6 | 1.00 | | 4 | 1.00 | |
| 2 | 48 | 0.80 | (0.5–1.3) | 18 | 2.37 | (0.9–6.6) | 10 | 1.25 | (0.4–4.3) |
| 3 | 16 | 0.54 | (0.3–1.0) | 5 | 1.24 | (0.3–4.7) | 4 | 0.86 | (0.2–3.9) |
| ⩾4 | 6 | 0.47 | (0.2–1.2) | 2 | 1.00 | (0.2–5.8) | 1 | 0.45 | (0.1–4.6) |
| Age at first full-term birth[d] | | | | | | | | | |
| <20 | 18 | 1.00 | | 6 | 1.00 | | 4 | 1.00 | |
| 20–24 | 28 | 0.89 | (0.5–1.7) | 6 | 0.32 | (0.1–1.2) | 5 | 0.60 | (0.2–2.2) |
| 25–29 | 32 | 1.11 | (0.6–2.2) | 10 | 0.99 | (0.3–3.2) | 6 | 0.66 | (0.2–2.5) |
| ⩾30 | 27 | 1.23 | (0.6–2.5) | 9 | 1.37 | (0.4–4.8) | 3 | 0.48 | (0.1–2.4) |
| Body mass index (kg $m^{-2}$) | | | | | | | | | |
| <22 | 61 | 1.00 | | 12 | 1.00 | | 8 | 1.00 | |
| 22–24.59 | 33 | 0.55 | (0.4–0.9) | 11 | 0.99 | (0.4–2.3) | 8 | 1.31 | (0.5–3.6) |
| 24.6–29.02 | 33 | 0.57 | (0.4–0.9) | 8 | 0.71 | (0.3–1.8) | 7 | 1.22 | (0.4–3.5) |
| ⩾29.03 | 25 | 0.41 | (0.2–0.7) | 10 | 0.92 | (0.4–2.3) | 5 | 0.52 | (0.1–1.9) |

[a] Includes 13 women diagnosed with both intraductal carcinoma and lobular carcinoma *in situ*, nine with cribriform carcinoma *in situ*, two with intraepithelial carcinoma *in situ*, one with Paget's disease of the nipple and four with unknown histology. [b] Relative risks adjusted for age at diagnosis study, site, smoking, number of mammograms in the 5 years prior to 1 year before reference date, and all other risk factors in this table. [c] Relative risks adjusted for age at diagnosis, site, smoking, number of mammograms in the 5 years prior to 1 year before reference date, and all other risk factors in this table except number of full-term births. [d] Among parous women only.

adjusted for the number of mammograms in the 5 year period prior to 1 year before reference date, but further adjustment for other screening methods (i.e. physical breast examination or BSE) did not alter the RRs, owing to the correlation between use of different screening methods. Local and regional/distant tumours were most likely to be detected by the patient or her partner (through BSE or accidental discovery), and our results are similar to those of a recent study of breast cancer patients in Wisconsin, where 22% of invasive tumours in premenopausal women were detected by routine mammograms and 72% by BSE or accidental discovery (Reeves *et al.*, 1995). A possible source of residual confounding arises from differing methods used to detect the tumours. To assess this potential confounding in the present study, further analyses were carried out using case data only (Begg and Zhang, 1994). Relative risks for models including a risk factor, screening history and other confounders were calculated for local and regional/distant tumours relative to *in situ* tumours. The addition of detection method in the model had little effect on the odds rations, giving no evidence of residual confounding by method of detection.

A history of breast cancer in a first-degree relative is an established risk factor, especially among younger women (Eby *et al.*, 1994), and in this study a greater than 2-fold risk was seen for each stage of diagnosis. The risks in the BCDDP study were slightly lower (RR = 1.5, Brinton *et al.*, 1983), possibly because the controls in that study had volunteered to be screened and may have had a higher prevalence of a family history of breast cancer than the general population. Previous studies have shown a greater risk of *in situ* compared with invasive tumours among patients with previous breast biopsies or benign breast disease (Brinton *et al.*, 1983; Dubin *et al.*, 1984; Claus *et al.*, 1993), possibly as a result of early detection through frequent screening. In the present study, the magnitude and significance of the increased risk associated with a breast biopsy was greater for *in situ* tumours than for local or regional/distant tumours, even after adjusting for number of mammograms. Benign breast disease is an established risk factor for invasive breast cancer, and women with atypical hyperplasia are at a particularly high risk (Bodian, 1993; Ma and Boyd, 1992). The greater association of biopsy with *in situ* tumours than with local or regional/distant tumours supports the close relationship between benign tumours, carcinoma *in situ* and invasive carcinoma (Bodian, 1993). It is also possible that the lack of a clear demarcation between atypical hyperplasia and *in situ* tumours may result in diagnostic misclassification, leading to the observed association (Bodian, 1993; Marcus *et al.*, 1994).

An increased breast cancer risk among young African–American women compared with white women remains largely unexplained (Kelsey and Horn Ross, 1993), and in this study the increase persisted after adjusting for possible confounders. The increased risk for *in situ* disease among African–Americans was also found in a case–control study of a screened population of women aged over 35, but, in contrast, no increase was seen for invasive disease (Dubin *et al.*, 1984).

Early age at menarche is an established risk factor for breast cancer (Kelsey *et al.*, 1993). There was some evidence of an increased risk with earlier age at menarche for local tumours, but no association with carcinoma *in situ.* The BCDDP study also found no association with *in situ* tumours or small tumours, but there was a significant increasing trend with younger age at menarche for tumours greater than 1 cm (Brinton *et al.*, 1983).

Nulliparity is also an established breast cancer risk factor, though the increased risk is not so apparent among women aged less than 40 (Janerich and Hoff, 1982; Kelsey *et al.*,

1993; Velentgas and Daling, 1994). The present study shows variation in the effect of parity by stage of disease. Nulliparous women were at a significantly increased risk of *in situ* and local tumours. Women with four or more full-term births were at about half the risk of women with a single birth, for both *in situ* and local disease. This reduction in risk has not been seen previously (Dubin *et al.*, 1984). In contrast, no clear effect of parity was seen for regional/distant tumours. One possible explanation for this difference is that if pregnancy causes a short-term increase in breast cancer risk followed by a long-term protection effect (Kelsey *et al.*, 1993), parous women aged less than 45 may have protection from early-stage tumours, but not from later stage tumours. However, one would then expect a decreasing trend in RR of *in situ* tumours with increasing time since last birth, and this was not apparent. Late age at first birth is a breast cancer risk factor, especially among younger women (Velentgas and Daling, 1994), and was significantly associated with regional/distant tumours and to a lesser extent, *in situ* and local tumours. The BCDDP study (Brinton *et al.*, 1983) showed a significant increase in risk of all stages of disease with increasing age at first birth, but another study showed no relationship between *in situ* tumours and age at first birth (Dubin *et al.*, 1984).

Consumption of a lifetime average of two or more alcoholic drinks per day was associated with a significantly increased risk of regional/distant tumours in this study. To our knowledge, the relationship between stage of breast cancer and alcohol consumption has not previously been examined, but other studies have found overall associations with alcohol consumption (Rosenberg *et al.*, 1993). The association has been seen in both cohort and case–control studies, and persists after adjustment for known confounding factors. If alcohol consumption is indeed a causal factor of breast cancer, it would be one of few readily modifiable risk factors known (the others being physical exercise in younger women and weight loss in older women; Brinton, 1994). However, no definite biological explanation for the association is known. There are several possible mechanisms including the stimulation of prolactin secretion, decreased clearance of oestrogen by the liver (Velentgas and Daling, 1994), or possibly an alcohol-induced increase in total oestrogen levels (Reichman *et al.*, 1993), and further research is needed in this area. The lack of a significant increased risk for *in situ* tumours found in the present study may be due to small numbers, or may indicate that alcohol affects the progression of tumours from *in situ* to invasive. Detailed analyses of alcohol intake in this study are currently underway.

Some studies have shown an inverse association between body mass index and breast cancer risk in premenopausal women (Hunter and Willet, 1993), and the relationship in the present study has been analysed previously (Swanson *et al.*, 1996). Small tumours are more difficult to detect in obese women, but the reduced risk of *in situ* and local tumours associated with increased BMI is unlikely to be due to detection bias since the inverse relationship held among women whose tumours were found by mammography, a detection method unlikely to be affected by BMI (Swanson *et al.*, 1996).

A previously published analysis of this data (Brinton *et al.*, 1995) has examined the RRs of different stages of breast cancer associated with use of oral contraceptives, and found that use for at least 6 months was associated with both local and regional/distant tumours, but not *in situ* tumours. This supports evidence from other studies (Kay and Hannaford, 1988; Romieu *et al.*, 1989; Olsson *et al.*, 1991) that oral contraceptives can induce cell proliferation or other late-stage events.

This study is one of the largest to examine risk factors by histological type of early-stage breast cancer and our results support the theory that ductal carcinoma *in situ* is more closely related to invasive breast cancer than the lobular form. Results from studies of risk factors by histological types of *in situ* breast cancer have been inconsistent (Marcus *et al.*, 1994). The association between family history and DCIS has been suggested previously (Erdreich *et al.*, 1980) but the present study is the first to show a significantly increased risk. Several previous studies (Rosen *et al.*, 1982; Claus *et al.*, 1993) have suggested that LCIS is related to family history. The 4-fold risk of LCIS following a previous breast biopsy is not unexpected, as LCIS is usually detected as a result of a biopsy given for some other reason (Bodian, 1993).

To conclude, this study provides epidemiological support for the theory that *in situ*, local and regional/distant breast cancer are closely related. Increased risks of similar magnitude for all stages of disease were associated with a family history of breast cancer. For some risk factors, including a previous breast biopsy, parity, African–American race and body mass index, the magnitude of association was greater for *in situ* disease than for local or regional/distant disease and this persisted after adjustment for number of mammograms, indicating that it was not due to screening bias. This tends to suggest that *in situ* tumours are likely to be on the causal pathway of invasive tumours. The significant association between alcohol consumption and invasive tumours, but not *in situ* tumours, indicates that alcohol may be involved in late-stage events. Analyses by histological type of *in situ* tumours suggested that both ductal and lobular carcinoma *in situ* were associated with most established breast cancer risk factors, and the magnitude of association tended to be greater for the ductal form.

**Acknowledgements**

We are grateful to Drs Tim Byers, Virginia Ernster, Jennifer Kelsey, Nancy Potischman, Bruce Stadel and Dimitrios Trichopoulos for invaluable input on the study design. Successful management of the project was due to the efforts of Florence Wilson and Betsy Bridgman in Atlanta, Tom English in New Jersey, and Diane Setterholm in Seattle, who worked with an extremely competent group of interviewers. The integrity of the data was further assured by the following individuals at Westat Inc.: Elizabeth Lovoy, Eric Mehl, Linea Efner and Diana Seybolt. Finally, we thank the many women who graciously agreed to participate in this study.

### References

ARMITAGE P AND BERRY G. (1987). *Statistical Methods in Medical Research*. pp. 205–207. Blackwell: Oxford.

BEGG CB AND ZHANG Z-F. (1994). Statistical analysis of molecular epidemiology studies employing case–series. *Cancer Epidemiol., Biomarkers & Prev.*, **3**, 19–24.

BODIAN CA. (1993). Benign breast disease, carcinoma *in situ* and breast cancer risk. *Epidemiol. Rev.*, **15**, 177–187.

BRINTON LA. (1994). Ways that women may possibly reduce their risk of breast cancer. *J. Natl Cancer Inst.*, **86**, 1371–1372.

BRINTON, LA, HOOVER R AND FRAUMENI JF. (1983). Epidemiology of minimal breast cancer. *JAMA.*, **249**, 483–487.

BRINTON LA, DALING JR, LIFF J, SCHOENBERG JB, MALONE KE, STANFORD JL, COATES RJ, GAMMON MD, HANSON L AND HOOVER RN. (1995). Oral contraceptives and breast cancer risk among younger women. *J. Natl Cancer Inst.*, **87**, 827–835.

CALUS EB, RISCH N, THOMPSON WD AND CARTER D. (1993). Relationship between breast histopathology and family history of breast cancer. *Cancer*, **71**, 147–153.

DIXON WJ (ed.) (1990). *BMDP Statistical Software Manual*. **2**, pp. 1047–1077. University of California Press: Berkeley.

DUBIN N AND PASTERNACK BS. (1986). Risk assessment for case–control subgroups by polychotomous logistic regression. *Am. J. Epidemiol.*, **123,** 1101–1117.

DUBIN N, HUTTER RVP, STRAX P, FAZZINI EP, SCHINELLA RA, BATANG ES AND PASTERNACK BS. (1984). Epidemiology of minimal breast cancer among women screened in New York City. *J. Natl Cancer Inst.*, **73,** 1273–1279.

EBY N, CHANG-CLAUDE J AND BISHOP DT. (1994). Familial risk and genetic susceptibility for breast cancer. *Cancer Causes Control*, **5,** 458–470.

ERDREICH LS, ASAL NR AND HOGE AF. (1980). Morphologic types of breast cancer: age, bilaterality, and family history. *Southern Med. J.*, **73,** 28–32.

FLEISS JL. (1973). *Statistical Methods for Rates and Proportions.* pp. 146–147. Wiley: New York.

FEUER EJ AND WUN L-M. (1992). How much of the recent risk in breast cancer incidence can be explained by increases in mammographic utilization? A dynamic modelling approach. *Am. J. Epidemiol.*, **136,** 1423–1436.

HANKEY BF, BRINTON LA, KESSLER LG AND ABRAMS J. (1993). Breast cancer. In *SEER Cancer Statistics Review 1973–1990.* Miller BA, Ries LAG, Hankey BF, Kosary CL, Harras A, Devesa SS and Edwards BJ. (eds.) pp. IV.1–IV.4. US National Cancer Institute NIH Pub. No. 93–2789: Washington DC.

HOLZMAN D. (1995). News. *J. Natl Cancer Inst.*, **87,** 710–711.

HUNTER DJ AND WILLET WC. (1993). Diet, body size and breast cancer. *Epidemiol. Rev.*, **15,** 110–132.

JANERICH DT AND HOFF M. (1982). Evidence of a crossover in breast cancer risk factors. *Am. J. Epidemiol.*, **116,** 737–742.

KAY CR AND HANNAFORD PC. (1988). Breast cancer and the pill–a further report from the Royal College of General Practioners' oral contraception study. *Br. J. Cancer*, **58,** 675–680.

KELSEY JL AND HORN ROSS PL. (1993). Breast cancer: Magnitude of the problem and descriptive epidemiology. *Epidemiol. Rev.*, **15,** 7–16.

KELSEY JL, GAMMON MD AND JOHN EM. (1993). Reproductive factors and breast cancer. *Epidemiol Rev.*, **15,** 36–47.

LANTZ PM, REMINGTON PL AND NEWCOMB PA. (1991). Mammography screening and increased incidence of breast cancer in Wisconsin. *J. Natl Cancer Inst.*, **83,** 1540–1546.

LIFF JM, SUNG JFC, CHOW WH, GREENBERG RS AND FLANDERS WD. (1991). Does increased detection account for the rising incidence of breast cancer? *Am. J. Pub. Health.*, **81,** 462–465.

MA L AND BOYD NF. (1992). Atypical hyperplasia and breast cancer risk: a critique. *Cancer Causes Control*, **3,** 517–525.

MARCUS JN, WATSON P, PAGE DL AND LYNCH HT. (1994). Pathology and heredity of breast cancer in younger women. *Monogr. Natl Cancer Inst.*, **16,** 23–34.

OLSSON H, RANSTAM J, BALDETORP B, EWERS SB, FERNO M, KELLANDER D AND SIGURDSSON H. (1991). Proliferation and DNA ploidy in malignant breast tumors in relation to early contraceptive use and early abortions. *Cancer*, **67,** 1285–1290.

PERCY C, VAN HOLTON H AND MUIR C. (eds) (1990). *International Classification of Diseases for Oncology.* pp. 132–133. World Health Organization: Geneva.

PONTEN J, HOLMBERG L, TRICHOPOLOUS D, KALLIONIEMI OP, KVALE G, WALLGREN A AND TAYLOR-PAPADIMITRIOU J. (1990). Biology and natural history of breast cancer. *Int. J. Cancer*, **(suppl. 5),** 5–21.

REEVES MJ, NEWCOMB PA, REMINGTON PL AND MARCUS PM. (1995). Determinants of breast cancer detection among Wisconsin (United States) women. *Cancer Causes Control*, **6,** 103–111.

REICHMAN ME, JUDD JT, LONGCOPE C, SCHATZKIN A, CLEVIDENCE BA, NAIR PP, CAMPBELL WS AND TAYLOR PR. (1993). Effects of alcohol consumption on plasma and urinary hormone concentrations in premenopausal women. *J. Natl. Cancer Inst.*, **85,** 722–727.

ROMIEU I, WILLETT WC, COLDITZ GA, STAMPFER MJ, ROSNER B, HENNEKENS CH AND SPEIZER FE. (1989). Prospective study of oral contraceptive use and risk of breast cancer in women. *J. Natl Cancer Inst.*, **81,** 1313–1321.

ROSEN PP, LESSER ML, SENIE RT AND KINNE DW. (1982). Epidemiology of breast carcinoma. III. Relationship of family history to tumour type. *Cancer*, **50,** 171–179.

ROSENBERG L, METZGER LS AND PALMER JR. (1993). Alcohol consumption and risk of breast cancer: a review of the epidemiologic evidence. *Epidemiol. Rev.*, **15,** 133–144.

SEER PROGRAM. (1983). *Summary Staging Guide, Cancer Surveillance Epidemiology and End Results Reporting.* US DHHS: Bethesda.

SWANSON CA, COATES RJ, SCHOENBERG JB, MALONE KE, GAMMON MD, STANFORD JL, SHORR IJ, POTISCHMAN NA AND BRINTON LA. (1996). Body size and breast cancer risk among women under age 45. *Am. J. Epidemiol.* (in press).

VELENTGAS P AND DALING JR. (1994). Risk factors for breast cancer in younger women. *Monogr. Natl Cancer Inst.*, **16,** 15–22.

WHITE E, LEE CL AND KRISTAL AR. (1990). Evaluation of the increase in breast cancer incidence in relation to mammographic use. *J. Natl Cancer Inst.*, **82,** 1546–1552.